# Modern Stents: Where Are We Going?

**DOI:** 10.5041/RMMJ.10403

**Published:** 2020-04-29

**Authors:** Ofer Kobo, Majdi Saada, Simcha R. Meisel, Elias Hellou, Aaron Frimerman, Rami Abu Fanne, Jameel Mohsen, Asaf Danon, Ariel Roguin

**Affiliations:** 1 Department of Cardiology, Hillel Yaffe Medical Center, Hadera, Israel; 2 The Ruth & Bruce Rappaport Faculty of Medicine, Technion–Israel Institute of Technology, Haifa, Israel

**Keywords:** Bare metal stent, drug-eluting stents, history, percutaneous coronary intervention

## Abstract

Coronary artery stenting is the treatment of choice for patients requiring coronary angioplasty. We describe the major advancements with this technology. There have been significant developments in the design of stents and adjunctive medical therapies. Newer-generation drug-eluting stents (DES) have almost negligible restenosis rates and, when combined with proper anti-platelet treatment and optimal deployment, a low risk of stent thrombosis. The introduction of newer-generation DES with thinner stent struts, novel durable or biodegradable polymer coatings, and new antiproliferative agents has further improved the safety profile of early-generation DES. In parallel the effectiveness has been kept, with a significant reduction in the risk of target lesion revascularization compared with the early-generation DES. However, to date, the development of completely bioresorbable vascular scaffolds has failed to achieve further clinical benefits and has been associated with increased thrombosis. Newer-generation DES—including both durable polymer as well as biodegradable polymer—have become the standard of care in all patient and lesion subsets, with excellent long-term results.

## INTRODUCTION

Cardiovascular diseases, and specifically ischemic heart disease, are among the leading causes of morbidity and mortality worldwide.^1^ Percutaneous coronary intervention (PCI) revolutionized the treatment of coronary artery disease, and it is nowadays the most common method of coronary revascularization.^2^

The first balloon angioplasty in the coronary arteries was performed in 1977 by Dr Andreas R. Gruentzig in Zurich, Switzerland. This angioplasty procedure utilized an expandable balloon, fashioned on a kitchen table in Gruentzig’s apartment. The efficacy of balloon angioplasty was limited by acute closure of the coronary artery in 5% to 10% of patients and high rates of restenosis, which occurred in as many as half of all revascularizations within the first year. Restenosis occurred by both elastic recoil and subsequent proliferation of smooth muscle cells at the site of endothelial damage caused by balloon inflations.^3^–^5^

Coronary stents were introduced in 1986 as an innovative solution and breakthrough for the acute recoil and vessel closure. The first stents were implanted by Puel in Toulouse and Sigwart in Zurich. Named the WALLSTENT, they were a stainless steel wire-mesh structure, self-expanding after deployment, and manufactured by Schneider AG (Bulach, Switzerland). At first, stents had high metallic density, resulting in a high incidence of sub-acute stent thrombosis. They were bulky and technically challenging to use, resulting in frequent failure in deployment and embolization.^6^,^7^

It took several years to make stent implantation safe by refining the adjusted pharmacology and improving technical aspects of the procedure. Only in 1994 were the first coronary stents approved by the US Food and Drug Administration (FDA). Dr Julio Palmaz, a radiologist, designed a balloon-expandable stainless-steel slotted metal tube, instead of a spring or coil. By trying different designs and types of metal, Dr Palmaz together with Dr Richard Schatz, a cardiologist, ultimately developed the Palmaz–Schatz stent. The stent was designed to provide a scaffold that would increase the acute gain in lumen diameter compared with percutaneous transluminal coronary balloon angioplasty alone and, thereby, reduce the rate of clinically relevant restenosis following PCI. The first FDA-approved stent had a mesh form built from stainless steel 163L and was relatively bulky.^8^,^9^

Since the Palmaz–Schatz stent, PCI has consistently evolved over time with the introduction of new and improved devices, techniques, and adjunctive pharmacotherapy.

## BARE-METAL STENTS

The first stents were metal stents and were initially used as a bailout strategy for complication during balloon angioplasty.^10^ Most of the stents were made from stainless steel and were balloon-expandable. They were mounted on a balloon and were deployed by balloon inflation. Some were self-expandable and made from nitinol, a thermal shape memory alloy composed of nickel and titanium. After the introduction of the drug-eluting stent (DES) in the early 2000s, the first stents became known as *bare metal stents* (BMS).

**Figure 1:**
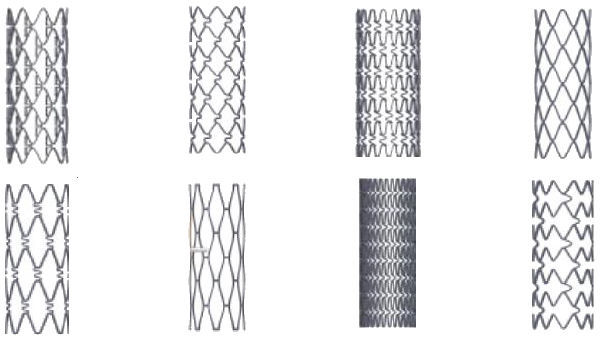
Stent Structures Several examples of the different geometrical stent structures in the early years of stents. Adapted from Figure 3 of Paisal et al.^13^ [CC by 3.0].

The superiority of BMS over balloon angioplasty was shown in several randomized controlled trials, and elective stenting became a feasible clinical option.^11^,^12^ The technology of BMS improved in order to increase flexibility, “pushability,” and radial strength. Better metal alloys were introduced (Table 1), as well as improved geometrical structures and better delivery systems (Figure 1). These improvements led to increased use of BMS over time. However, despite all improvements, BMS still resulted in early and late stent adverse events.^14^ The main challenge of BMS was high in-stent restenosis rates due to neointimal hyperplasia, which occurred in 10%–30% of all stents. Predictors for stent failure included clinical factors, such as diabetes, and procedural and angiographic factors, such as treatment of bifurcation lesions, longer stents, small-caliber stents, stents that were not well opposed to the vessel wall, and lack of intracoronary imaging. Intravascular ultrasound initially was critical to understanding that stents were not properly being deployed, and its use identified the need for high-pressure inflations. This subsequently allowed the shift to antiplatelet agents and enabled patients to be discharged either the next day or, now, the same day. Prior to this observation patients had to be anticoagulated, which required prolonged hospitalization. Some authors suggested that routine use of intravascular imaging may result in better long-term outcomes.^8^

**Table 1:** Composition of Stent Alloys (Weight Percentage).

Material	Fe	Co	Cr	Pt	Ni	W	Mo	Mn	Ti
316L SS	63		18		14		2.6	<2.0	
CoCr (L605)	3	50.5	20		10	15		1.5	
PtCr	37		18	33	9		2.6		
Nitinol					55				45

Co, cobalt; Cr, chromium; Fe, iron; Mn, manganese; Mo, molybdenum; Ni, nickel; Pt, platinum; SS, stainless steel; Ti, titanium; W, tungsten.

Many different strategies tried to address the issue of restenosis, for example heparin-coating of the stents or endothelial-seeded stents. Intracoronary beta and gamma radiation was also used for a brief period, but it caused endothelial damage and reduced ability for local tissue healing. This technology was almost completely abandoned, and is still being used in only few centers worldwide.

Among the many solutions was the idea that antiproliferative drugs could be delivered locally via a polymer release mechanism in high enough concentrations to reduce neointimal hyperplasia but at a slow enough rate to avoid systemic toxicity. It was this insight that allowed the development of DES.

## DRUG-ELUTING STENTS

First approved in the early 2000s, DES shared the stainless-steel backbone of BMS. However, they had new components: an immunosuppressant or cytotoxic drug to inhibit neointimal hyperplasia and a polymer to which that drug was fixed. The purpose of the polymer was to serve as a diffusion barrier that allowed the prolonged release of the antineoplastic agent.^15^ Use of DES had significantly improved clinical outcomes as compared with BMS, primarily through a notable reduction in the rates of repeat revascularization.

The first generation of DES released sirolimus and paclitaxel over the course a month, resulting in a significant reduction in the need for revascularization compared with BMS.^16^,^17^ The number needed to treat to prevent repeat revascularization was between 6 and 10, according to a large meta-analysis. First-generation DES soon became the mainstay of PCI. However, it was suggested in 2006 that DES may carry an increased non-negligible risk for late stent thrombosis, compared to BMS.^18^,^19^ Extensive analyses failed to confirm this concern, but safety improvements were studied and implemented. The potent anti-restenosis effect of early-generation DES came at the expense of delayed arterial healing of the stented coronary segment, characterized by chronic inflammation at the stented site with uncovered stent struts, coronary evaginations and positive vessel remodeling, fibrin deposition, and neoatherosclerosis.^20^ This pathological process has been proposed as a mechanism of the risk of late thrombotic events—specifically very late stent thrombosis after implantation of the early-generation Cypher sirolimus-eluting stent and Taxus paclitaxel-eluting stent.

**Figure 2:**
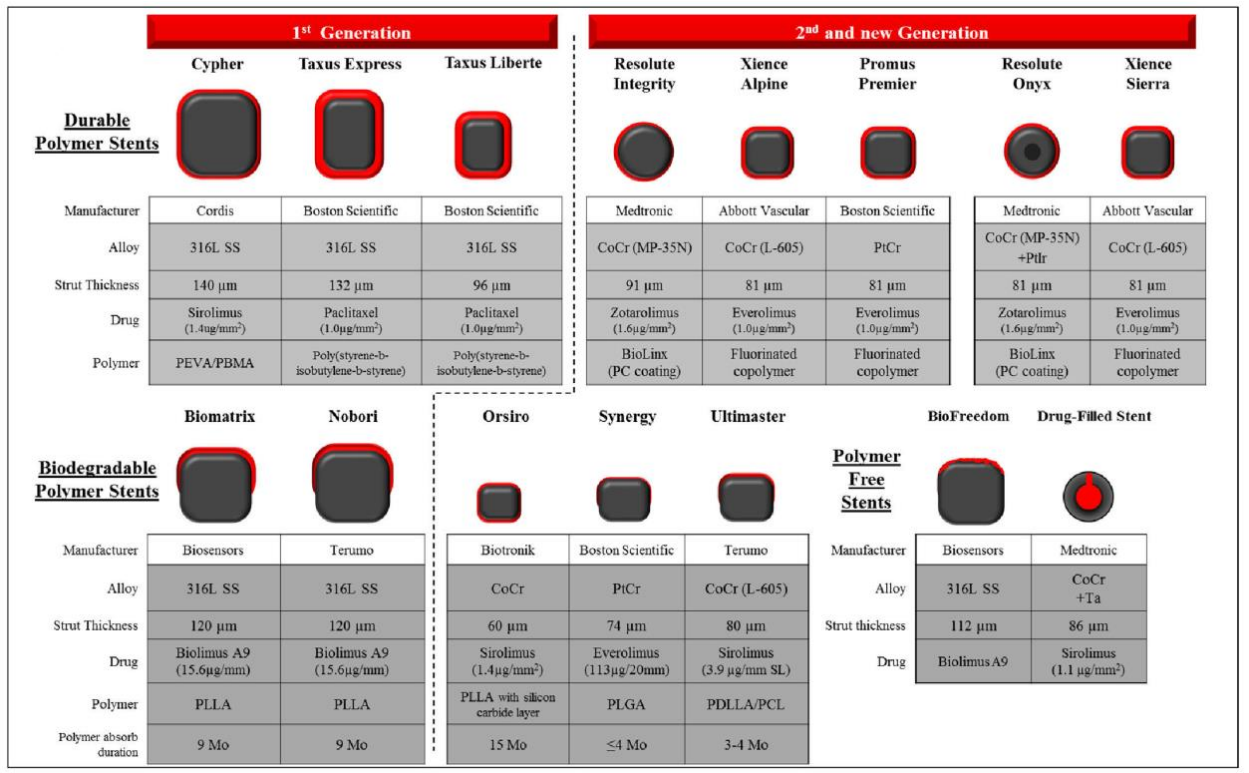
Different DES Designs Design characteristics (cross-sectional cut) of representative drug-eluting stents and bioabsorbable scaffold/stents. The characteristics of past and current commercial drug-eluting stents including durable polymer (DP)-, biodegradable polymer (BP)-, and polymer free-DES. Types of materials (alloy, drug, and polymer), strut thickness, and estimated duration of polymer absorption (in BP-DES) for each stent are described. CoCr, cobalt chromium; Ir, iridium; Mo, months; PBMA, poly(butyl methacrylate); PC, phosphorylcholine-coated; PCL, poly-ɛ-caprolactone; PDLA, poly-d-lactic acid; PDLGA, poly(d,l-lactide-co-glycolide); PDLLA, poly-d,l-lactic acid; PEVA, poly (ethylene-vinyl acetate); PGA, polyglycolic acid; PLGA, poly(lactide-co-glycolide); PLLA, poly-l-lactic acid; Pt, platinum; SS, stainless steel.

In order to improve DES performance, and to achieve the ideal characteristics of flexibility, trackability, radial strength, and biocompatibility, constant efforts were invested in improvement of all the components—the platform, polymer, and drug. Second-generation DES have an improved platform with thinner strut thickness, allowing faster healing and endothelialization of the coronaries, and less inflammation and injury to the media as well. The platform was made of cobalt-chromium instead of stainless steel to make it more flexible and deliverable. Additionally, fluorinated polymers were developed that were more biocompatible and had thrombo-resistant properties. The eluted drugs in the second-generation DES were also changed, with the use of rapamycin derivatives with improved safety profile. Different DES designs are shown in Figure 2. Studies found that the duration of the dual antiplatelet therapy (DAPT) was very important in preventing stent thrombosis, especially for first-generation DES.^22^,^23^

A large randomized controlled trial (RCT) comparing second-generation DES and BMS found, over 6 years of follow-up, reduced risk of repeat revascularization and definite stent thrombosis, with the use of DES. It should be noted, however, that no improvement in mortality was proven.^24^ A pooled, patient-level analysis of four RCTs comparing second- and first-generation DES over a follow-up period of 2 years found significant reduction in the rates of stent thrombosis, with a concomitant reduction in cardiac death or myocardial infarction (MI)^22^; these results were confirmed in a further meta-analysis.^23^

Even with contemporary DES, and despite the significant reduction in thrombotic events, late stent failure is a concern.^20^,^25^,^26^ Some mechanisms were suggested to cause late stent failure, including hypersensitivity reaction, stent fracture, and in-stent neoatherosclerosis. Unlike atherosclerosis in native coronary arteries which develops over decades, in-stent neoatherosclerosis is a rapid process that may occur in months to years following stent placement.^27^ The incidence of neoatherosclerosis is similar after first- and second-generation DES implantation.^20^ Other potential causes for DES failure may be polymer-related. During stent implantation, polymers are at risk of bonding, webbing, cracking, or peeling. This may provide a thrombogenic nidus and decrease the uniformity of drug delivery, which may lead to stent thrombosis or restenosis.^28^ Recent developments target these mechanisms, with the aim to reduce and eliminate the long-term stent failure; new developments include polymer-free stents, bioresorbable polymer (BP) DES, and fully bioresorbable stents—commonly known as bioresorbable scaffolds.

## POLYMER-FREE DES

The development of polymer-free DES was aimed at preventing adverse events caused by hypersensitivity reactions to polymer. Another potential problem these stents meant to solve is the cracking of the polymer coating while the stent is being inflated in the coronary artery. As the polymer purpose is to modify and control drug release, the challenge of polymer-free DES is to adequately control drug dose and elution kinetics in the early period after stent implantation. This required modification of the stent structure or the drug used. One suggested solution is a drug-filled stent (DFS) designed to provide controlled drug elution from an internal stent lumen without a polymer coating. In early trials the DFS showed encouraging clinical outcomes, minimal neointimal hyperplasia, and a high degree of stent strut coverage at 1 month post implantation optical coherence tomography (OCT).^29^ The use of a lipophilic rapamycin analogue, biolimus A9, in a polymer-free DES showed clinical benefits in patients at high risk of bleeding.^30^

A meta-analysis of 16 RCTs, with a total of over 15,000 patients, was recently published, comparing polymer-free and polymer-coated DES. Polymer-free DES were associated to a significant 18% reduction in total mortality (OR 0.82, 95% CI 0.68–0.99); however, no significant difference in main adverse cardiovascular events (MACE) was noted. An adequate subgroup analysis was not performed due to lack of data.^31^

## BIORESORBABLE POLYMER DES

The hypothesis that led to of the development of bioabsorbable polymer was that chronic inflammatory responses to the polymer enhances late DES failure. Therefore, once the polymer dissolves, the stimulus for chronic inflammation will be eliminated. The biodegradable polymer matrix is composed of either polylactic or polylactic-co-glycolic acid. These polymers are converted to carbon dioxide and water, typically between 6 weeks and 24 months, depending on polymer configuration. Several large-scale studies proved the safety of the bioresorbable polymer (BP) DES and its non-inferiority over contemporary DES.^32^,^33^ A large meta-analysis including over 80,000 patients compared the biodegradable polymer biolimus-eluting stent to durable polymer drug-eluting and bare-metal stents, and found BP-DES to have a worse safety profile compared to contemporary DES.^34^ However, a more contemporary meta-analysis, including several BP-DES, found BP-DES to have similar safety and efficacy profiles to second-generation durable polymer DES.^35^

An ultrathin-strut, bioabsorbable-polymer, sirolimus-eluting stent (OrSiro, Biotronik, Bulach, Switzerland) showed clinical benefit over second-generation DES, mainly in acute coronary syndrome,^36^,^37^ and its superiority in ST elevation myocardial infarction patients was also concluded from a large real-life PCI registry.^38^ Whether this suggested benefit is a BP-DES class effect or is secondary to the ultrathin struts formation is yet to be proven.

## BIORESORBABLE VASCULAR SCAFFOLDS

What if the implanted stent would open the narrowing, keep the artery open for the period needed to heal, and then disappear? This is the logic behind the fully bioresorbable stents or scaffolds.

In-stent neoatherosclerosis occurs in BMS as well as in first- and second-generation DES.^20^ The development of fully bioresorbable vascular stents or scaffolds (BVS) aimed to reduce the incidence of this phenomenon. Other potential benefits of BVS include restoration of vasomotor function of the stented segment, elimination of the possibility of late stent fracture, improving side branch survival, and reducing the limitation of subsequent surgical revascularization of the stented segment. Some patients may also prefer avoiding a foreign body, if given the choice.^39^ The BVS consists of synthetic biodegradable polymers that are intended to initially provide the benefits of DES and then dissolve within months after implantation. However, in order to provide the mechanical benefits of DES, thicker stent struts are always the prerequisite for BVS.

The first RCT comparing BVS to contemporary DES, the ABSORB III trial, demonstrated the everolimus-eluting poly-l-lactic acid-based Absorb bioresorbable vascular scaffold (BVS) to be non-inferior to everolimus-eluting stents.^40^ However, despite the positive outcomes, at 3-year follow-up, BVS were associated with significantly higher incidence of stent thrombosis and target-vessel MI.^41^ Further disappointing results for the BVS derived from the AIDA RCT. The trial data and safety monitoring recommended early reporting, which revealed significantly higher definite or probable device thrombosis with bioresorbable scaffolds (HR 3.87, 95% CI 1.78–8.42).^42^

The hypothesis of improved vascular healing process with the use of BVS was also questioned. A trial using OCT for 6- and 12-month follow-up after implantation of BVS and DES found BVS to have a lower rate of uncovered and/or non-apposed struts; however, evaginations and discontinuities in device was more frequent with BVS.^43^

Recently, the 5-year follow up of the ABSORB III trial was published, with a landmark analysis after 3 years (time of device absorption). The period of excess risk for BVS ended at 3 years, coincident with complete scaffold resorption.^44^ It was also suggested that improved implantation technique may improve the clinical outcomes of the BVS^45^; nonetheless, in view of the current clinical data, BVS are not recommended for clinical use outside the setting of clinical studies.^46^

Newer BVS technologies and devices are currently undergoing clinical and preclinical testing. One of the more promising BVS is a magnesium-based resorbable scaffold. Compared to previously used polymer, magnesium alloys possess somewhat better mechanical properties and biocompatibility as stent materials.^47^ Imaging studies of the device revealed benign healing process at the edges of the BVS at 12-month follow-up.^48^ However, to date, the clinical data on this device are limited.

Pooled analysis of 184 patients and 189 lesions from the BIOSOLVE II and BIOSOLVE III trials revealed favorable efficacy and safety profiles during up to 12 months of follow-up, with no definite or probable scaffold thrombosis observed.^49^ Further clinical trials of the magnesium-based BVS with larger cohorts and longer follow-up are ongoing.

While the clinical evidence for the magnesium-based BVS is appealing, further development of zinc-based BVS has been carried out. Preliminary studies demonstrated that a tailored zinc-based material could be a promising candidate for a better stent material in the future.^47^ Currently, although some scaffolds have the CE mark and are sold in Europe, most clinicians use them only in the setting of clinical studies.^46^

## DRUG-COATED BALLOONS

Another technology is to apply the anti-proliferative drug medication (usually paclitaxel) to a balloon. The medication is attached to the balloon using several different coating methods. Using a prolonged 60-second inflation, mainly in sites of restenosis, the medication is delivered locally to the tissue. The results of treating restenosis are similar to implanting a second layer of DES. Ample research is being performed to understand the place of drug-coated balloons in the treatment *de novo* lesions, mainly in small-caliber vessels.^50^ Currently the price of drug-coated balloons is much higher than DES, and their use is limited.

## CONCLUSIONS

**Figure 3:**
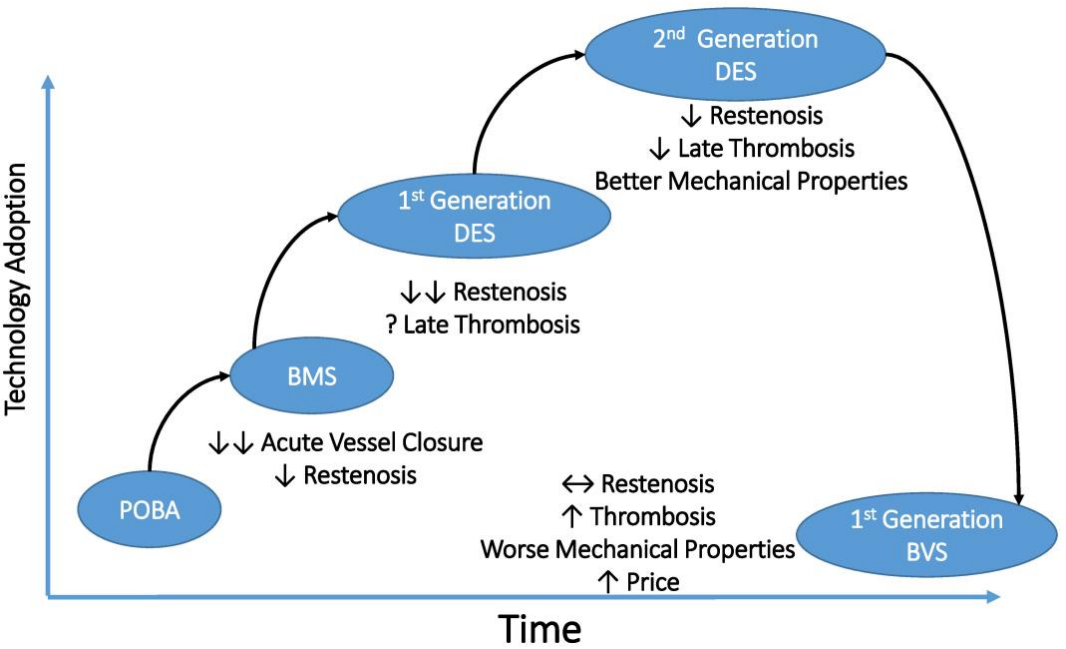
Evolution of Coronary Stents BMS, bare metal stent; BVS, bioresorbable vascular scaffolds; DES, drug-eluting stent; POBA, plain balloon angioplasty; ↔, balanced; ↑, increased; ↓, decreased; ↓↓, greatly decreased.

Coronary artery stenting is the treatment of choice for patients requiring coronary angioplasty. There have been significant developments in the design of BMS platforms, leading to reduction in restenosis. However, the newer-generation DES have almost negligible restenosis rates and, when combined with DAPT and optimal deployment, a low risk of stent thrombosis. The introduction of newer-generation DES with thinner stent struts, novel durable or biodegradable polymer coatings, and new antiproliferative agents has further improved the safety profile of early-generation DES. In parallel, the effectiveness was kept, with a significant reduction in the risk of target lesion revascularization compared with the early-generation DES. Accordingly, newer-generation DES—including both durable polymer and biodegradable polymer—have become the standard of care in all patient and lesion subsets, with excellent long-term results. The development of BVS, however, has failed to achieve further clinical benefits and is associated with increased thrombosis (Figure 3).

There are a number of ongoing studies to evaluate newer stent platforms, anti-proliferative drugs, novel polymers, polymer-free stents, and bioresorbable stents. It may be challenging for any new stent design or material to demonstrate better effectiveness and still be worth the cost of innovation. Interventional cardiologists have in their arsenal a wide variety of stents available with excellent performance. The quest for the ideal stent continues, but it will take a very large study in order to achieve this and prove device superiority.

## Abbreviations

BMS: bare metal stent
BVS: bioresorbable vascular stents/scaffolds
DES: drug-eluting stents
MACE: main adverse cardiovascular events
OCT: optical coherence tomography
PCI: percutaneous coronary intervention
